# Association of Intranasal and Neurogenic Dural Inflammation in Experimental Acute Rhinosinusitis

**DOI:** 10.3389/fphar.2020.586037

**Published:** 2020-10-15

**Authors:** Luka Lovrenčić, Ivica Matak, Zdravko Lacković

**Affiliations:** Laboratory of Molecular Neuropharmacology, Department of Pharmacology, University of Zagreb School of Medicine, Zagreb, Croatia

**Keywords:** facial pain expressions, headache, neurogenic inflammation, cFos protein, rhinosinusitis

## Abstract

**Background:**

Nasal cavity and sinus disorders, such as allergic rhinitis, rhinosinusitis, or certain anatomical defects, are often associated with transient or ongoing headaches. On the other hand, migraine headache patients often exhibit pain referral over the area of nasal sinuses and typical nasal autonomic symptoms involving congestion and rhinorrhea. Mechanism for convergence of nasal or sinus disorders and headaches is unknown. Herein, we examined the association of sino-nasal inflammatory pain with common preclinical indicators of trigeminovascular system activation such as dural neurogenic inflammation (DNI) and neuronal activation in brainstem nociceptive nuclei.

**Methods:**

Nasal and paranasal cavity inflammation and pain was induced by formalin (2.5%/10 μl) or capsaicin (0.1%/10 μl) instillation at the border of maxillary sinus and nasal cavity in rats. Quantification of inflammation of nasal mucosa and DNI was performed by spectrophotometric measurement of Evans blue - plasma protein complex extravasation. Pain behavior was quantified by rat grimace scale (RGS). Nociceptive neuronal activation in caudal part of spinal trigeminal nucleus (TNC) was assessed by c-Fos protein immunohistochemistry.

**Results:**

Capsaicin and formalin administered into rat nasal cavity increased plasma protein extravasation in the nasal mucosa and dura mater. Intensity of plasma protein extravasation in nasal mucosa correlated with extravasation in dura. Similarly, facial pain intensity correlated with nociceptive neuronal c-Fos activation in the TNC.

**Conclusion:**

Present data show that inflammatory stimuli in deep nasal and paranasal structures provoke distant intracranial changes related to trigeminovascular system activation. We hypothesize that this phenomenon could explain overlapping symptoms and comorbidity of nasal/paranasal inflammatory disorders with migraine.

## Introduction

Transient headache is a common symptom of different sino-nasal disorders, which is usually resolved by successful treatment of the underlying condition. Nasal and sinus inflammatory conditions, some anatomic abnormalities such as septal spine, bullous nasal turbinate and intranasal contact points have been found to be associated with migraine (Lee et al., 2017). Sino-nasal disorders, such as sinusitis, allergic rhinitis, and mixed rhinitis, could be associated with higher prevalence of migraine and other headaches (Ku et al., 2006; Martin et al., 2014; Wang et al., 2016). Migraine patients may have more intranasal contact points between opposing mucosal surfaces (Ferrero et al., 2014) and their operative removal leads to migraine improvement in some patients. Ongoing headache associated with pain referral above the area of sinuses and accompanied by autonomic symptoms in the sino-nasal area such as nasal congestion, rhinorrhea, and lacrimation has been previously referred to as “sinus headache”. However, according to current third edition of International Headache Society Classification criteria (ICHD-3), this term is outdated since it encompasses both primary headaches such as migraine (which may be presenting with nasal symptoms), and secondary headache attributed to disorders of the nose or sinuses. In absence of typical inflammatory findings, many patients previously diagnosed by themselves or by the physician with “sinus headache” are eventually diagnosed with migraine (Schreiber et al., 2004; Eross et al., 2007), and treatable with antimigraine drugs (Patel et al., 2013). Also, in animal models, various stimuli used to model migraine (mechanical or electrical sagittal sinus stimulation, chemical stimulation of meninges) (Mitsikostas and Sanchez del Rio, 2001) and nasal inflammation induce c-Fos expression in the caudal part of spinal trigeminal nucleus (TNC) (Anton et al., 1991). Overall, the role of nasal pathology in the pathogenesis of migraine remains controversial.

In present study, we assessed the possibility that painful inflammatory stimuli in the sino-nasal area are associated with neurogenic inflammation of cranial meninges and neuronal activation of second order nociceptive sensory neurons, the markers of trigeminovascular system activation.

## Materials and Methods

### Animals

Male Wistar rats 2 to 3 months old and weighing 280 to 400 g, bred at the Department of Pharmacology at the Faculty of Medicine of the University of Zagreb, kept at 12-h light/dark cycle with free access to food and water were used in all experiments. The experiments were planned and conducted in accordance with European Union Directive (2010/63/EU) and International Association for the Study of Pain (Zimmermann, 1983). The experiments were approved by Ethical Committees of University of Zagreb School of Medicine and Croatian Ministry of Agriculture (permission no. EP 03-2/2015).

### Chemicals

Formalin (formaldehyde mass concentration 36%) (T.T.T., Sveta Nedjelja, Croatia) was diluted with saline (0.9% sodium chloride solution) to the 2.5% concentration required for intranasal instillation. The concentration of formalin used was based on 2.5% concentration usually employed in the orofacial formalin test.

Capsaicin (Sigma Aldrich, St. Louis, MO, USA) was first diluted with ethanol to the mass concentration of 20% and kept at −20°C until further use. For the animal treatment, the solution was further diluted to 0.1% capsaicin concentration in 0.9% physiological saline. The concentration of capsaicin used was based on concentrations and doses previously used for subcutaneous stimulation of the rat hind paw (Bach-Rojecky and Lacković, 2005; Bach-Rojecky et al., 2008; Arreola-Peralta et al., 2018) and rat or mouse whisker pad (Pelissier et al., 2002; Rossi et al., 2016).

### Animal Treatment

**Figure 1:**
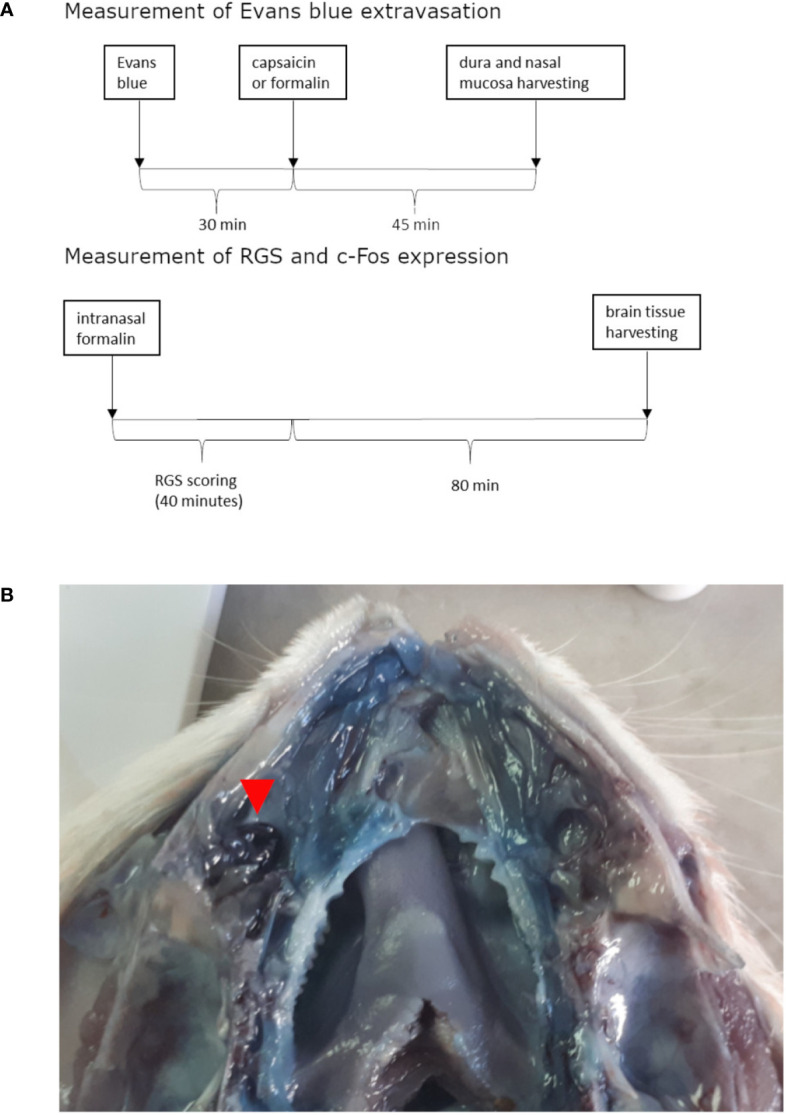
Timeline of experiments **(A)** and site of irritant instillation (in this case formalin) shown on opened nasal cavity (septum was removed) of rat injected with Evans blue and perfused with saline **(B)**. Formalin instillation site is situated near the entrance to the paranasal sinus and is marked with the red arrow. Near it marked Evans blue extravasation can be observed when comparing to contralateral side.

The animals were treated intranasally with formalin or capsaicin 30 min after 40 mg/kg Evans blue intravenous administration *via* the tail vein (for timeline see **Figure 1A**). Upon administration of Evans blue and irritants, the rats were briefly anesthetized with isoflurane inhalation (Forane, Baxter, Deerfield, IL, USA, 5% induction). Rats were taken out of the induction chamber, and prior to awaking, the volume of 10 μl was instilled by a plastic tubing (outer diameter 0.6 mm) coupled to Hamilton syringe (Hamilton 705 LT 50 µl, Hamilton Company, Reno, Nevada, USA), which was advanced approximately 1.5 cm through the nostril into the nasal cavity without significant resistance (for site of instillation see **Figure 1B**). The period needed for rat recovery from isoflurane anesthesia allowed enough time for instillation. Volume of 10 μl was employed based on the micro-computer tomography-calculated volume of maxillary sinus (Phillips et al., 2009). In preliminary experiments in saline-perfused animals, 10-μl methylene-blue injection induced ipsilateral localized coloration of the nasal mucosa near the entrance of the maxillary sinus (not shown).

### Quantification of Pain by Rat Grimace Scale

Twelve rats were used for determining the relationship between painful behavior assessed by rat grimace scale (RGS) (Sotocinal et al., 2011), and the neuronal activation in the primary trigeminal nociceptive nucleus, the TNC. The rats were divided in three experimental groups: non-treated (three animals), intranasally treated with saline (four animals), and the pain group intranasally treated with formalin (five animals). The number of animals used was determined according to previous studies (Matak et al., 2014; Lacković et al., 2016). In order to administer formalin, the animals were briefly restrained since we observed lack of behavioral response in animals subjected to inhalational anesthesia. In conscious animals, using a thin plastic tube coupled to Hamilton syringe was not appropriate due to duration of the procedure. Thus, to enable a quicker delivery, here we employed a flexible electrophoretic gel loading tip (tip diameter = 0.5 mm) coupled to a 10–100 µl Eppendorf pipette. Immediately following the 10-μl formalin instillation, the animals were put into transparent plastic cages for observation (for timeline of experiment see **Figure 1A**). For RGS scoring, the rat faces were captured with a digital camera in 3-min intervals for 39 min after formalin instillation. Three-minute intervals were chosen based on original description of the method where one photograph from each 3-min interval was chosen for scoring (Sotocinal et al., 2011). Rat facial expression was scored as previously described (Sotocinal et al., 2011). In brief, four action units related to whisker positioning, disappearance of the nose bulging and cheek crease, orbital tightening, and distinct ear shape and positioning, were analyzed. To each of this action units, score of 0, 1 (moderate), or 2 (severe) was assigned and the total sum of scores calculated. The experimenter who scored the individual images was blinded to the animal treatment.

### Neuronal c-Fos Activation

Of 12 RGS-scored rats, 10 were used for immunohistochemical analysis (2 non-treated, 4 saline treated, 4 formalin treated). Survival time between the intranasal instillation of formalin and the perfusion for immunohistochemistry was 2 h, as previously used in the orofacial formalin-evoked c-Fos expression (Matak et al., 2014). Animals were deeply anesthetized with 70 mg/kg ketamine (Richter Pharma AG, Wels, Austria) and 7 mg/kg xylazine (Alfasan, Woerder, Netherlands), thoracotomized and transcardially perfused with 500-ml saline and 250-ml fixating agent (4% paraformaldehyde in phosphate buffered saline (PBS)). After craniotomy, brains were collected and stored in 15% sucrose in fixating agent until tissue sank, followed by 30% sucrose in PBS for 24 h. Then, the tissue was taken out of sucrose, and frozen (-80°C). Caudal brainstem area containing TNC was embedded in O.C.T. compound (Tissue-Tek, Sakura Finetek, Japan) and cut to 30-μm coronal sections in the cryostat. Immunohistochemical staining for c-Fos was performed as described previously (Matak et al., 2014). In brief, representative slices of TNC were placed into the free-floating wells containing PBS and 0.25% Triton X-100 (Sigma-Aldrich, St. Louis, MO, USA) (PBST) and further rinsed with fresh PBST (3 × 5 min). To block the non-specific immunoreactivity, samples were incubated in 10% normal goat serum (Sigma-Aldrich, St. Louis, MO, USA) in PBST for 60 min. Then, the slices were incubated overnight at room temperature with the primary c-Fos antibody (sc-52, Santa Cruz, Dallas, TX, USA) (1:500 in PBST + 1% NGS). Next day, after rinsing 3 times with PBST, slices were incubated with a fluorescently labelled Alexa Fluor 488-labeled secondary antibody (Invitrogen, Carlsbad, CA, USA) for 2 h in the dark at room temperature. After 3 rinses with PBST, slices were mounted on slides, and covered with anti-fading coverslip agent (Fluoromount, Sigma-Aldrich, St. Louis, MO, USA). Sections were photographed by fluorescent microscope (Olympus BX-51, Olympus, Tokyo, Japan) with digital camera (Olympus DP-70, Olympus, Tokyo, Japan). Images were obtained in RGB color format, and grey images of separated green channel were further used to count the c-Fos-positive neuronal profiles, by employing associated software (cellSens Dimension, Olympus, Tokyo, Japan). The counting was performed by a blinded observer based on fixed threshold intensity parameters and defined minimal size of the objects. In tissue cuts, the number of objects was counted in the left-ipsilateral and right-contralateral side within observable borders of the TNC. Average number of c-Fos-expressing neuronal profiles was calculated based on four slices analyzed per single animal.

### Quantification of Dural and Nasal Inflammation

Nineteen rats that were used for experiment determining relationship between nasal and dural inflammation were administered Evans’ blue into tail vein and were divided into three experimental groups, each group intranasally received saline (six animals), capsaicin (six animals), or formalin (seven animals). The number of animals was determined according to previous work done by our group regarding dural Evans blue extravasation (Filipović et al., 2014) and was in accordance with simple sample size calculation for animal studies (Charan and Biswas, 2013). Forty-five minutes after intranasal injection, animals were deeply anesthetized with 70 mg/kg ketamine (Richter Pharma AG, Wels, Austria) and 7 mg/kg xylazine (Alfasan, Woerder, Netherlands), thoracotomized and transcardially perfused with 500-ml saline. After craniotomy, dura mater and nasal mucosa were harvested. To obtain the supratentorial dura primarily innervated by the trigeminal nerve, the dura mater was carefully separated from the skull base and neurocranium. Samples were weighed and incubated in 2-ml formamide (Honeywell, Muskegon, Michigan, USA) for 48 h on 37°C. The absorbances of formamide extracts of Evans blue were measured by spectrophotometer (Iskra, Ljubljana, Slovenia) set to 620 nm wavelength. Evans blue concentration in the tissue (ng/mg) was calculated based on the calibration curve and tissue weight.

### Statistical Analysis of Results

Results are presented as mean ± SEM and analyzed by one-way analysis of variance (ANOVA) followed by Newman Keuls *post hoc* test (p < 0.05 considered significant). Pearson’s correlation coefficient (r) and linear regression were used for the correlation analysis of variables (p < 0.05 considered significant).

## Results

### Pain Assessment by RGS Score

**Figure 2:**
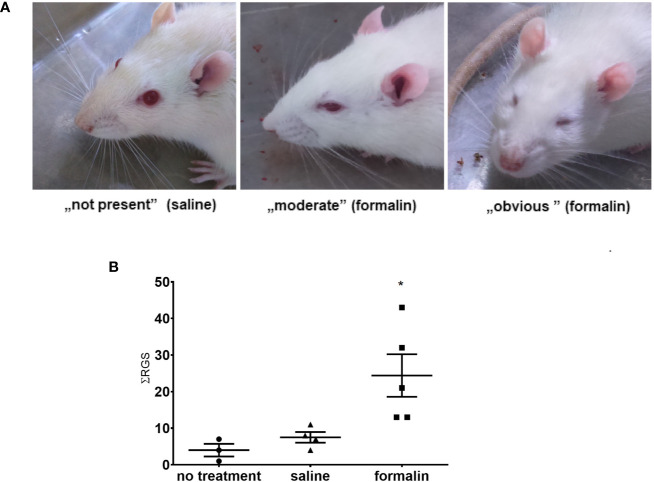
Stimulation of nasal mucosa with 2.5% formalin induces painful grimacing in rats. **(A)** The rat faces showing, from left to right, normal appearance (saline-treated animal), and different severity of painful facial expression in formalin-treated animals (moderate to obvious). In the images, left to right, orbital tightening is the best visible action with following rat grimace scale (RGS) scores ascribable to them: 0, 1, 2. **(B)** Animals treated with formalin had higher sum of total RGS scores (ƩRGS) assessed every 3 min during the 39 min period after intranasal treatment with formalin, compared to saline or no treatment (3–5 animals per group, mean ± SEM, * p < 0.05 in comparison to non-treated or saline-treated group, one-way ANOVA followed by Newman Keuls *post hoc* test).

After intranasal administration of formalin, the rats showed observable changes in the facial appearance suggestive of pain (**Figure 2A**), evident as increased total sum of RGS scores during 39 min after the formalin treatment (**Figure 2B**). Animals treated with saline did not show increased sum of RGS compared to non-treated rats.

### The c-Fos Expression and Its Association With RGS Score

**Figure 3:**
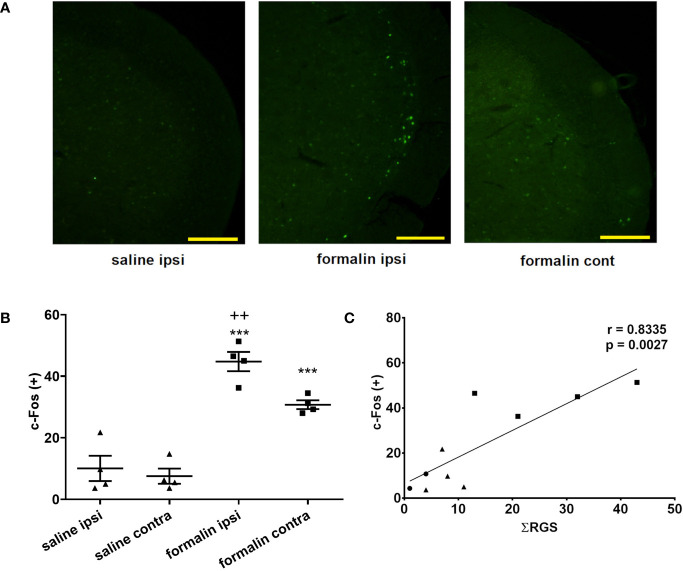
Intranasal formalin (2.5%, 10 μl) induces bilateral neuronal activation in the caudal part of spinal trigeminal nucleus (TNC), which correlates with painful facial grimacing behavior. **(A)** Neuronal activation was quantified by c-Fos immunohistochemistry, and subsequent automatic quantification of the number of c-Fos-expressing [c-Fos (+)] neuronal profiles in ipsilateral (ipsi) and contralateral (cont) TNC. **(B)** The images and quantification are representative of 4 slices per animal, 4 animals per treatment group. Scale bar = 200 µm. Data are represented as mean ± SEM. ***p < 0.001 compared to saline ipsilaterally or contralaterally, ^++^p < 0.01 compared to formalin contralaterally, ANOVA followed by Newman Keuls *post hoc* test). **(C)** The painful facial grimacing assessed by rat grimace scale total score, measured every 3-min intervals during 39-min observation period (Ʃ RGS) is significantly correlated with the number of c-Fos positive cells in the ipsilateral TNC (circle, no treatment; square, saline; triangle, formalin; r, Pearson’s correlation coefficient).

Animals treated with formalin had higher number of c-Fos-expressing neuronal profiles in the TNC compared to control (no treatment group) or saline (**Figure 3A**). Increased c-Fos expression was observed bilaterally after unilateral nasal formalin instillation. The intensity of c-Fos staining (**Figure 3A**, not quantified directly), and the number of c-Fos-expressing neurons (**Figure 3B**) was lower in the contralateral TNC. Average number of c-Fos positive cells per slice of TNC was 8 in non-treated animals which is similar to number in saline-treated animals.

In individual animals, there was a significant correlation between the sum of RGS scores following instillation of saline or formalin and the number of c-Fos positive cells in the ipsilateral TNC (**Figure 3C**). Similarly, there was a significant correlation of sum of RGS scores with number of c-Fos-expressing cells in the contralateral TNC (Pearson’s correlation coefficient r = 0.8164; p = 0.004) or the total number of c-Fos-expressing cells in bilateral TNC (Pearson’s r = 0.8295; p = 0.003) (results not shown).

### Inflammation of the Nasal Mucosa Is Associated With Dural Neurogenic Inflammation

**Figure 4:**
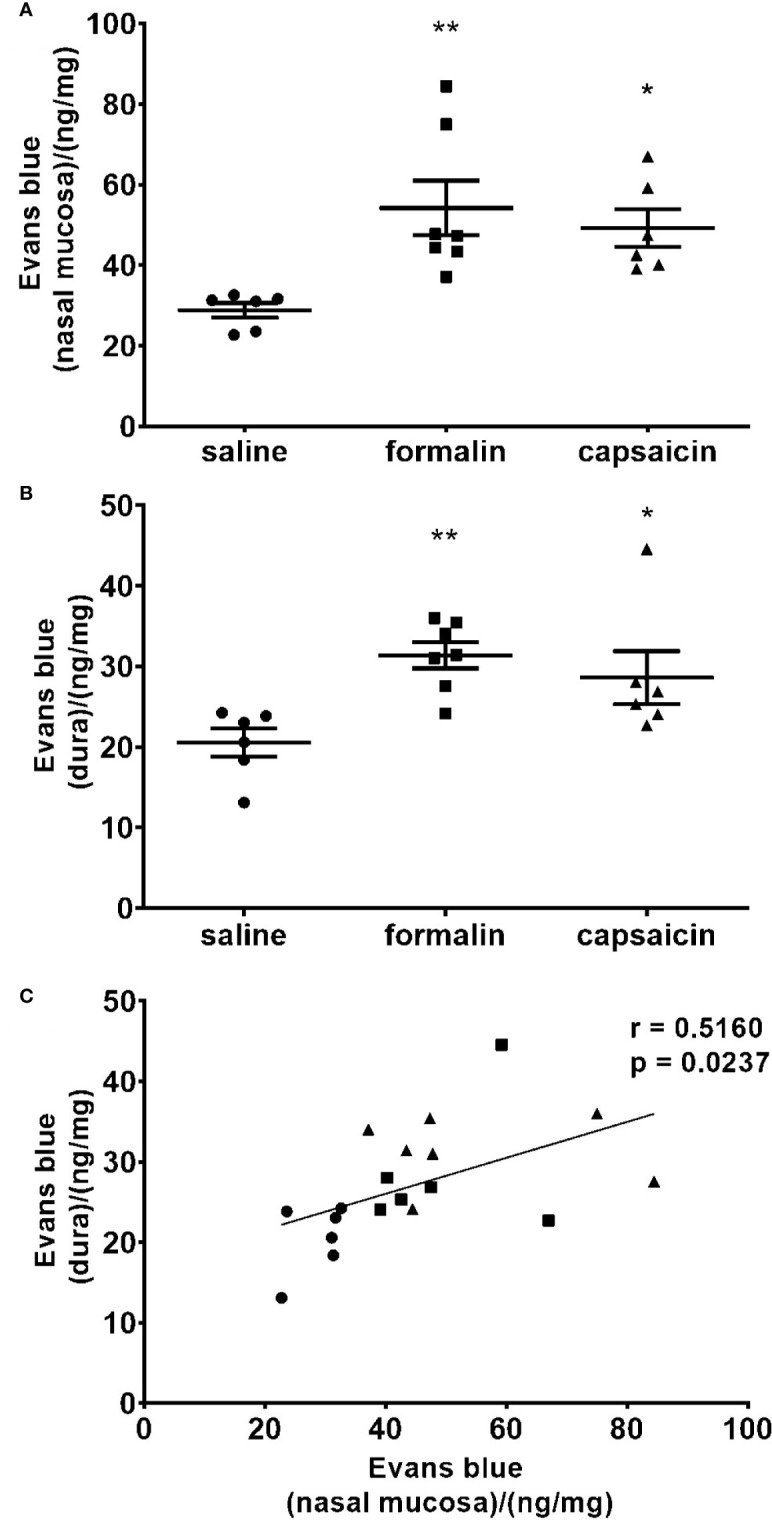
Local stimulation of the deep nasal mucosa by formalin (2.5%) or capsaicin (0.1%) induce plasma protein extravasation in the nasal mucosa and cranial dura mater. The inflammation was quantified by spectrophotometric measurement of formamide extracts of Evans blue dye (ng of the dye per mg of tissue in the nasal mucosa **(A)** and dura mater **(B)**. Mean ± SEM, 6–8 animals per group, *p < 0.05, **p < 0.01 vs. saline-treated group (one-way ANOVA followed by Newman Keuls *post hoc* test). Intensity of the inflammation in nasal mucosa correlated with the intensity of inflammation in cranial dura. **(C)** Individual animal values are plotted showing extravasation in the nasal mucosa (horizontal axis) and dura (vertical axis) for all experimental groups together (circle, saline; square, formalin; triangle, capsaicin; r, Pearson’s correlation coefficient).

There was a significantly increased tissue content of Evans blue in the nasal mucosa in the animals treated with intranasal capsaicin and formalin, indicative of increased plasma protein extravasation in comparison to saline treatment (**Figure 4A**). Extravasation of Evans blue was similarly elevated in the dura mater of rats treated with formalin or capsaicin relative to the control group treated with saline (**Figure 4B**) and shown to be significantly correlated with the Evans blue extravasation in nasal mucosa (**Figure 4C**). While harvesting dura, we did not observe any differences in Evans blue extravasation in dura on the side of irritant instillation compared to the contralateral side.

## Discussion

### Formalin-Induced Pain in the Sino-Nasal Area

Here, we observed that low dose formalin instillation into the deeper part of nasal cavity situated at the border of maxillary sinus induces nociceptive behavior, quantified behaviorally by RGS (**Figure 2**). Quantification of pain-related facial expression by RGS is a relatively new method for assessing experimental pain in rats, introduced in 2011 as an adaptation of mouse grimace scale (Sotocinal et al., 2011). Up to now, it has successfully been employed for postsurgical (Kawano et al., 2014; Waite et al., 2015), orthodontic (Liao et al., 2014), neuropathic (Akintola et al., 2017; Philips et al., 2017), and inflammatory pain (Asgar et al., 2015). Herein, we attempted to use additional methods of pain assessment, by measuring the duration of facial wiping and facial mechanical thresholds by Von Frey filaments. After formalin stimulation, we observed that only some of the animals exhibited sequences of facial wiping similar to the ones exerted in orofacial formalin test, which proved unreliable for pain assessment (results not shown). In addition, the animals did not exhibit alteration in cutaneous mechanical thresholds assessed with Von Frey filaments, suggestive of the lack of mechanical allodynia. This could be due to the site of irritant stimulation located deeply into the nasal cavity near the entrance into the maxillary sinus. Stimulation of deep orofacial structures, protected by bone mass, did not lead to pericranial allodynia or wiping behavior characteristic of experimental pain in more superficial pericranial structures. This suggests that, in the orofacial area, RGS scoring could be a method of choice when measurement of spontaneous rubbing behavior or mechanically-evoked pain response is unreliable/not present.

### Association of RGS With Brain c-Fos Activation

TNC is a relay nucleus for transduction of cranial pain, and its neurons express c-Fos following the acute noxious stimulation in the trigeminal innervation area (Hathaway et al., 1995), including the nasal mucosa (Anton et al., 1991). Up to now, the c-Fos expression in the TNC after irritant stimulation of the nasal mucosa by mustard oil (Anton and Peppel, 1991) and capsaicin (Plevkova et al., 2010) was shown to occur in anesthetized animals, i.e., without the behavioral assessment of pain. The c-Fos expression in the TNC combined with behavioral data was previously used in other nociceptive assays such as orofacial formalin test (Matak et al., 2014) and found to be in line with the behavioral effect of different pharmacological analgesic treatments.

Interestingly, we found that unilateral stimulation of nasal cavity and sinuses leads to bilateral activation of c-Fos in the TNC (**Figure 3**). In contrast to that, unilateral whisker pad stimulation in orofacial formalin test evokes unilateral c-Fos expression (Matak et al., 2014). Both sites of inflammatory stimulation (whisker pad formalin injection and unilateral nasal instillation) are very close to the medial line which represents the border between the innervation areas of left and right trigeminal nerves (Panneton et al., 2006). In present study, preliminary testing of the injection method with methylene blue dye do not support possible spread of the injected volume far from the instillation site, or a contralateral inflammation (**Figure 1B**). Thus, the reason for bilateral occurrence of c-Fos after unilateral nasal stimulation might be other than the direct stimulation of contralateral structures.

So far, the validation of RGS with biomarkers of pain has been scarce. In a model of orthodontic pain, RGS score was shown to be correlated to calcitonin gene-related peptide (CGRP) (Long et al., 2015), however, not with acid sensing ion channel 3 (ASIC3) periodontal expression (Gao et al., 2016). Correlation of RGS score with the neuronal activation in TNC (**Figure 3C**) further adds to validity of RGS as reliable method for pain assessment.

### Nociceptive Stimuli in Sino-Nasal Area Induce Dural Neurogenic Inflammation

In present experiments, we found that inflammatory stimulation of nasal mucosa by either formalin or capsaicin induces plasma protein extravasation in the cranial dura (**Figure 4**). Present results indicate a link between painful stimuli in the nasal/paranasal area, and the activation of dural afferents leading to dural neurogenic inflammation (DNI), which is commonly associated with headache.

Previous observations suggested that various types of irritation of the nasal mucosa lead to increased meningeal blood flow in anesthetized animals. Based on the investigation of umbellulone, a known headache trigger from shrub/tree plant *Umbellularia californica*, it was proposed that environmental irritants might either diffuse and directly activate meningeal afferents, or activate reflex pathways by stimulation of trigeminal endings in the nasal mucosa (Nassini et al., 2012; Kunkler et al., 2014). In present experiments, instillation of formalin or capsaicin unilaterally into the border between maxillary sinus and nasal cavity induces unilateral, localized inflammation, which correlated with the intensity of DNI. Thus, our findings are more in line with indirect activation of meningeal afferents by stimulation of a more limited number of sensory endings situated in the nasal/paranasal area, rather than the direct diffusion of irritants into the cranial meninges. One of the possible mechanisms for activation of meningeal afferents after stimulation of nasal mucosa might be the axonal reflex, since dura and nasal cavity share sensory innervation from common maxillary and ophthalmic branches of the trigeminal nerve, such as the ethmoidal nerve (Panneton et al., 2006; Poussel et al., 2012). Other mechanism could be the intraganglionic cross- communication between nearby primary sensory neurons which project to the intranasal structures and neurons which innervate the meninges (Kunkler et al., 2014). Third mechanism involving the cross-sensitization of second order sensory neurons within the TNC might also be involved. Neurons that conduct dural pain sensation relay to the same secondary neurons in the TNC as the neurons that conduct the pain sensation from the nasal cavity (Ellrich et al., 1999; Messlinger and Ellrich, 2001). The facts that we did not observe any gross differences in Evans blue extravasation in dura on the side of irritant instillation compared to the contralateral side and that previous models of infraorbital nerve constriction and formalin injection in vibrissal pad have shown similar Evans blue extravasation in dura ipsilateral to the injury and contralateral dura (Filipović et al., 2012) support cross-sensitization at the level of brainstem since brainstem is most likely site where signal could propagate to the side contralateral to the injury. Mentioned mutually non-exclusive mechanisms of convergence of sensory input could be responsible for increased incidence of primary headache in patients suffering from nasal and sinus conditions. Regardless of the level of convergence, activated dural nerves release neuropeptides (CGRP, VIP, substance P, neurokinin A, and PACAP-38) into dura which then cause neurogenic inflammation, vasodilation and increased vessel permeability (Vécsei et al., 2014).

Recently, we found that different types of experimental pain in trigeminal region induce DNI: chemically induced pain (formalin), neuropathic pain (infraorbital nerve constriction), inflammatory pain (CFA-evoked inflammation of temporomandibular joint) (Filipović et al., 2012; Filipović et al., 2014; Lacković et al., 2016). Neuropathic pain outside of cranial area (evoked by constriction or partial transection of sciatic nerve) did not result in DNI of cranial or spinal meninges (Filipović et al., 2014). In cranial meninges, the DNI was attenuated by anti-migraine drugs such as botulinum neurotoxin type A (BoNT/A) and sumatriptan (Filipović et al., 2012; Lacković et al., 2016). In addition, we found a reduction of CGRP expression in the cranial dura by pericranially injected BoNT/A as well as the colocalization of enzymatic products of BoNT/A and CGRP (Lacković et al., 2016). The involvement of CGRP is also suggested by observed efficacy of 5-HT1B/D agonist sumatriptan in reducing both pain and DNI (Lacković et al., 2016). These observations suggest that DNI is not associated only with migraine pathophysiology, but rather a non-specific event related to different types of pain in the trigeminal region.

Results presented here show that acute inflammation in the deep nasal area can induce trigeminovascular changes associated with headache. Understandably, bearing in mind the chronic nature of migraine and different disorders of nasal and sinus cavity, present findings derived by employing an acute stimulation model cannot be interpreted as a direct explanation for the possible pathophysiological link. However, the findings point to a possibility that long-term inflammation in the nasal/sinus area may induce more chronic changes leading to facilitated activation of the trigeminovascular system. It remains to be further investigated whether, in turn, the phenomenon of coupling of nasal inflammation with trigeminovascular activation is involved in observed comorbidities of acute or chronic paranasal and sinus disorders and headache.

## Conclusion

Present results suggest that acute nasal inflammatory stimulation correlates with painful nociceptive neuronal activation and neurogenic inflammation of cranial meninges, suggesting possible link between the painful disorders of nasal/paranasal craniofacial area and headaches.

## Data Availability Statement

The raw data supporting the conclusions of this article will be made available by the authors, without undue reservation.

## Ethics Statement

The animal study was reviewed and approved by Ethical Committees of University of Zagreb School of Medicine and Croatian Ministry of Agriculture (permission no. EP 03-2/2015).

## Author Contributions

ZL conceptualized experiments. IM and LL planned and performed the experiments. LL and IM analyzed data. All authors contributed to the article and approved the submitted version.

## Funding

This work was supported by Croatian Science Foundation (Project ID: IP-2014-09-4503) and University of Zagreb Support project.

## Conflict of Interest

The authors declare that the research was conducted in the absence of any commercial or financial relationships that could be construed as a potential conflict of interest.

## References

[B1] Akintola T. Raver C. Studlack P. Uddin O. Masri R. Keller A. (2017). The grimace scale reliably assesses chronic painin a rodent model of trigeminal neuropathic pain. Neurobiol. Pain 2 (October), 13–17. 10.1016/j.ynpai.2017.10.001 29450305 PMC5808980

[B2] Anton F. Peppel P. (1991). Central projections of trigeminal primaryafferents innervating the nasal mucosa: A horseradish peroxidase study in the rat. Neuroscience 41 (2–3), 617–628. 10.1016/0306-4522(91)90354-Q 1714553

[B3] Anton F. Herdegen T. Peppel P. Leah J. D. (1991). c-FOS-like immunoreactivity in rat brainstemneurons following noxious chemical stimulation of the nasal mucosa. Neuroscience 41 (2–3), 629–641. 10.1016/0306-4522(91)90355-R 1908066

[B4] Arreola-Peralta L. D. Altamirano-Reyna F. Galindo-González D. M. Solis-Anguiano J. G. Lacivita E. Leopoldo M. (2018). Potentiation of capsaicin-induced neurogenic inflammation by 5-HT7 receptors in the rat hind paw: Involvement of calcitonin gen-related peptide. Peptides 105 (April), 1–6. 10.1016/j.peptides.2018.05.002 29730242

[B5] Asgar J. Zhang Y. Saloman J. L. Wang S. Chung M.-K. Ro J. Y. (2015). The role of TRPA1 in muscle pain and mechanicalhypersensitivity under inflammatory conditions in rats. Neuroscience 310, 206–215. 10.1016/j.neuroscience.2015.09.042 26393428 PMC4633371

[B6] Bach-Rojecky L. Lacković Z. (2005). Antinociceptive effect of botulinum toxin type A in rat model of Carrageenan and Capsaicin induced pain. Croat. Med. J. 46 (2), 201–208. 15849840

[B7] Bach-Rojecky L. Dominis M. Lacković Z. (2008). Lack of anti-inflammatory effect of botulinumtoxin type A in experimental models of inflammation. Fundam. Clin. Pharmacol. 22 (5), 503–509. 10.1111/j.1472-8206.2008.00615.x 18717739

[B8] Charan J. Biswas T. (2013). How to calculate sample size for different studydesigns in medical research? Indian J. Psychol. Med. 35 (2), 121–126. 10.4103/0253-7176.116232 24049221 PMC3775042

[B9] Ellrich J. Andersen O. K. Messlinger K. Arendt-Nielsen L. (1999). Convergence of meningeal and facial afferentsonto trigeminal brainstem neurons: An electrophysiological study in rat and man. Pain 82 (3), 229–237. 10.1016/S0304-3959(99)00063-9 10488673

[B10] Eross E. Dodick D. Eross M. (2007). The sinus, allergy and migraine study(SAMS). Headache 47 (2),213–224. 10.1111/j.1526-4610.2006.00688.x 17300361

[B11] Ferrero V. Allais G. Rolando S. Pozzo T. Allais R. Benedetto C. (2014). Endonasal mucosal contact points in chronicmigraine. Neurol. Sci. 35 (SUPPL. 1), 83–87. 10.1007/s10072-014-1749-x 24867843

[B12] Filipović B. Matak I. Bach-Rojecky L. Lacković Z. (2012). Central action of peripherally applied botulinumtoxin type a on pain and dural protein extravasation in rat model of trigeminal neuropathy. PloS One 7 (1), 1–8. 10.1371/journal.pone.0029803 PMC3251614 22238656

[B13] Filipović B. Matak I. Lacković Z. (2014). Dural neurogenic inflammation induced byneuropathic pain is specific to cranial region. J. Neural Transm. 121 (5), 555–563. 10.1007/s00702-013-1144-4 24366531

[B14] Gao M. Long H. Ma W. Liao L. Yang X. Zhou Y. (2016). The role of periodontal ASIC3 in orofacial paininduced by experimental tooth movement in rats. Eur. J. Orthod. 38 (6), 577–583. 10.1093/ejo/cjv082 26675477

[B15] Hathaway C. B. Hu J. W. Bereiter D. A. (1995). Distribution of fos-like immunoreactivity in thecaudal brainstem of the rat following noxious chemical stimulation of the temporomandibular joint. J. Comp. Neurol. 356 (3), 444–456. 10.1002/cne.903560311 7642805

[B16] Kawano T. Takahashi T. Iwata H. Morikawa A. Imori S. Waki S. (2014). Effects of ketoprofen for prevention of postoperative cognitive dysfunction in aged rats. J. Anesth. 28 (6), 932–936. 10.1007/s00540-014-1821-y 24676769

[B17] Ku M. Silverman B. Prifti N. Ying W. Persaud Y. Schneider A. (2006). Prevalence of migraine headaches in patientswith allergic rhinitis. Ann. Allergy Asthma Immunol. 97 (2), 226–230. 10.1016/S1081-1206(10)60018-X 16937756

[B18] Kunkler P. E. Ballard C. J. Pellman J. J. Zhang L. J. Oxford G. S. Hurley J. H. (2014). Intraganglionic signaling as a novelnasal-meningeal pathway for TRPA1-dependent trigeminovascular activation by inhaled environmental irritants. PloS One 9 (7), 1–10. 10.1371/journal.pone.0103086 PMC4117521 25077949

[B19] Lacković Z. Filipović B. Matak I. Helyes Z. (2016). Activity of botulinum toxin type A in cranialdura: implications for treatment of migraine and other headaches. Br. J. Pharmacol. 173 (2), 279–291. 10.1111/bph.13366 26493010 PMC5341233

[B20] Lee M. Erickson C. Guyuron B. (2017). Intranasal pathology in the migraine surgerypopulation: incidence, patterns, and predictors of surgical success. Plast. Reconstr. Surg. 139 (1), 184–189. 10.1097/PRS.0000000000002888 28027245

[B21] Liao L. Long H. Zhang L. Chen H. Zhou Y. Ye N. (2014). Evaluation of pain in rats through facialexpression following experimental tooth movement. Eur. J. Oral. Sci. 122 (2), 121–124. 10.1111/eos.12110 24428464

[B22] Long H. Liao L. Gao M. Ma W. Zhou Y. Jian F. (2015). Periodontal CGRP contributes to orofacial painfollowing experimental tooth movement in rats. Neuropeptides 52, 31–37. 10.1016/j.npep.2015.06.006 26164378

[B23] Martin V. T. Fanning K. M. Serrano D. Buse D. C. Reed M. L. Bernstein J. A. (2014). Chronic rhinitis and its association withheadache frequency and disability in persons with migraine: Results of the American Migraine Prevalence and Prevention (AMPP) Study. Cephalalgia 34 (5), 336–348. 10.1177/0333102413512031 24275145

[B24] Matak I. Rossetto O. Lacković Z. (2014). Botulinum toxin type A selectivity for certaintypes of pain is associated with capsaicin-sensitive neurons. Pain 155 (8), 1516–1526. 10.1016/j.pain.2014.04.027 24793910

[B25] Messlinger K. Ellrich J. (2001). Meningeal nociception: Electrophysiologicalstudies related to headache and referred pain. Microsc. Res. Tech. 53 (2), 129–137. 10.1002/jemt.1077 11301488

[B26] Mitsikostas D. D. Sanchez del Rio M. (2001). Receptor systems mediating c-fos expressionwithin trigeminal nucleus caudalis in animal models of migraine. Brain Res. Rev. 35 (1), 20–35. 10.1016/S0165-0173(00)00048-5 11245884

[B27] Nassini R. Materazzi S. Vriens J. Prenen J. Benemei S. De Siena G. (2012). The “headache tree” via umbelluloneand TRPA1 activates the trigeminovascular system. Brain 135 (2), 376–390. 10.1093/brain/awr272 22036959

[B28] Panneton W. M. Gan Q. Juric R. (2006). Brainstem projections from recipient zones ofthe anterior ethmoidal nerve in the medullary dorsal horn. Neuroscience 141 (2), 889–906. 10.1016/j.neuroscience.2006.04.055 16753263

[B29] Patel Z. M. Kennedy D. W. Setzen M. Poetker D. M. Delgaudio J. M. (2013). “Sinus headache”: Rhinogenicheadache or migraine? An evidence-based guide to diagnosis and treatment. Int. Forum Allergy Rhinol. 3 (3), 221–230. 10.1002/alr.21095 23129234

[B30] Pelissier T. Pajot J. Dallel R. (2002). The orofacial capsaicin test in rats: Effects ofdifferent capsaicin concentrations and morphine. Pain 96 (1–2), 81–87. 10.1016/S0304-3959(01)00432-8 11932064

[B31] Philips B. H. Weisshaar C. L. Winkelstein B. A. (2017). Use of the rat grimace scale to evaluate neuropathic pain in a model of cervical radiculopathy. Comp. Med. 67 (1), 34–42. 28222837 PMC5310623

[B32] Phillips J. E. Ji L. Rivelli M. A. Chapman R. W. Corboz M. R. (2009). Three-dimensional analysis of rodent paranasal sinus cavities from X-ray computed tomography (CT) scans. Can. J. Vet. Res. 73 (3), 205–211. 19794893 PMC2705075

[B33] Plevkova J. Poliacek I. Antosiewicz J. Adamkov M. Jakus J. Svirlochova K. (2010). Intranasal TRPV1 agonist capsaicin challengeand its effect on c-fos expression in the guinea pig brainstem. Respir. Physiol. Neurobiol. 173 (1), 11–15. 10.1016/j.resp.2010.05.015 20580681

[B34] Poussel M. Varechova S. Demoulin B. Chalon B. Schweitzer C. Marchal F. (2012). Nasal stimulation by water down-regulates coughin anesthetized rabbits. Respir. Physiol. Neurobiol. 183 (1), 20–25. 10.1016/j.resp.2012.05.021 22659128

[B35] Rossi H. L. Broadhurst K. A. Luu A. S. Lara O. Kothari S. D. Mohapatra D. P. (2016). Abnormal trigeminal sensory processing in obesemice Heather. Pain 157 (1), 235–246. 10.1097/j.pain.0000000000000355 26397933 PMC4933294

[B36] Schreiber C. P. Hutchinson S. Webster C. J. Ames M. Richardson M. S. Powers C. (2004). Prevalence of migraine in patients with ahistory of self-reported or physician-diagnosed “sinus” headache. Arch. Intern. Med. 164 (16), 1769. 10.1001/archinte.164.16.1769 15364670

[B37] Sotocinal S. G. Sorge R. E. Zaloum A. Tuttle A. H. Martin L. J. Wieskopf J. S. (2011). The Rat Grimace Scale: A partially automatedmethod for quantifying pain in the laboratory rat via facial expressions. Mol. Pain 7 (1), 55. 10.1186/1744-8069-7-55 21801409 PMC3163602

[B38] Vécsei L. Tuka B. Tajti J. (2014). Role of PACAP in migraineheadaches. Brain 137 (3), 650–651. 10.1093/brain/awu014 24549810

[B39] Waite M. E. Tomkovich A. Quinn T. L. Schumann A. P. Dewberry L. S. Totsch S. K. (2015). Efficacy of Common Analgesics for Postsurgical Pain in Rats. J. Am. Assoc. Lab. Anim. Sci. 54 (4), 420–425. 26224443 PMC4521577

[B40] Wang I. C. Tsai J. D. Lin C. L. Shen T. C. Li T. C. Wei C. C. (2016). Allergic rhinitis and associated risk ofmigraine among children: A nationwide population-based cohort study. Int. Forum Allergy Rhinol. 6 (3), 322–327. 10.1002/alr.21654 26446370

[B41] Zimmermann M. (1983). Ethical guidelines for investigations of experimental pain in conscious animals. Pain 16 (2), 109–110. 10.1016/0304-3959(83)90201-4 6877845

